# Patient-centered supportive roadmap: patients and caregivers need to drive their care

**DOI:** 10.1093/icvts/ivaf033

**Published:** 2025-03-08

**Authors:** Cecilia Pompili, Jill Feldman, Shani Shilo, Jonathan Koffman

**Affiliations:** Institute for Clinical and Applied Health Research, Thoracic Surgery Unit, University of Hull, Hull, UK; Lung Cancer Advocate and EGFR Resister co-founder; The Israeli Lung Cancer Foundation, Rehovot, Israel; Wolfson Palliative Care Research Centre, University of Hull, Hull, UK

**Keywords:** palliative care, lung cancer advocacy, patient-centred care

We read with great interest Temel *et al*.’s research [1] demonstrating a palliative stepped-care model that incorporates declines in patient-reported quality of life (QoL) as a trigger for more intensive palliative care. This approach reduces the frequency of palliative care visits while maintaining patient-reported QoL. These findings hold significant potential for patients with lung cancer, including those with early-stage disease. Early palliative care has often been likened to an ‘umbrella’ kept at hand for the proverbial rain [2]. However, how can we ensure that this form of protection is equally relevant to patients with early-stage disease or those who are not undergoing prolonged treatment regimens?

In the context of radically treated disease, many patients who are not hospitalized or undergoing active regimens may still struggle with post-treatment sequelae, often for many months, including prolonged physical and psychophysical side effects. These may be inadequately self-managed or managed by those in primary and non-specialized care. This is particularly evident for post-surgical symptoms, including shortness of breath and wound infections, which result in more emergency department visits and impact the overall QoL [3]. Patients are less likely to discuss these symptoms with their surgical team. However, when they do so, this leads to better clinical outcomes [4]. Deterioration of their health status after surgery may also preclude additional systemic complementary treatments, thus affecting their clinical outcomes.

Monitoring QoL can help identify patients who might benefit from more active management of symptoms and treatment-related side effects and complications. While oncologists may now be more attuned to the benefits of early palliative care, we believe this awareness is still not adequately recognized by the surgical community. Historical and cultural preconceptions linking palliative care with the cessation of active treatment continue to pose barriers. The American College of Surgeons has now highlighted this gap by formally recognizing palliative care as an essential component of surgical education, underscoring its importance in assessing and alleviating patient suffering.

## PATIENTS’ PERSPECTIVES

While much of the focus of palliative care has been on advanced-stage disease and active treatment, patients with early-stage lung cancer who have completed curative intent face persistent and often unaddressed challenges. Shortness of breath, chronic fatigue and lingering treatment-related side effects can profoundly impact daily life as well as emotional distress and anxiety, leading to frustration, isolation and decreased emotional well-being [5]. For example, something as simple as climbing stairs or attending a social event can become daunting. These challenges are compounded by the lack of comprehensive follow-up care and support services that address the unique needs of lung cancer survivors.

Addressing these unmet needs with evidence-based, patient-centred approaches—like stepped palliative care—could significantly improve outcomes across the cancer continuum. Tailored palliative care, integrated early and guided by quality-of-life assessments, ensures that patients receive the right level of support at the right time. For patients who are no longer actively treating their disease, this approach validates their ongoing struggles. It acknowledges that survivorship is more than being disease-free—it is about living well despite cancer’s lasting impact. Such care models offer hope for better symptom management and improved QoL for patients and their families.

## CAREGIVERS’ PERSPECTIVES

The diagnosis of lung cancer is a devastating experience for the patient. Fortunately, most patients have a caregiver, who may be a spouse, parent, child, close friend or other family member [6]. Caregivers, especially those who live with the patient, embark on their own journey—one that involves numerous responsibilities. These include managing the patient’s overall care, navigating bureaucratic processes, providing emotional and physical support during symptoms and side effects, maintaining the household, supporting other family members and, above all, staying strong for everyone involved [7, 8]. The QOL and well-being of the patient have a direct impact on the QOL and well-being of the caregiver. Therefore, improving the patient’s QOL benefits not only the patient but also the caregiver and the entire household.

Advances in personalized medicine and earlier detection of lung cancer have led to increased survivorship and prolonged life expectancy, significantly extending the journeys of both the patient and the caregiver. This highlights the growing importance of providing extended supportive and palliative care during families’ journeys.

Temel *et al*. demonstrated that a stepped palliative care model was as effective as an early palliative care model, offering a more scalable approach to delivering palliative care worldwide. This finding is critical, as improving the patient’s QOL can positively impact the QOL of all family members living with the patient. It is essential to improve tracking of symptoms and side effects to ensure palliative care is provided to those who need it, ultimately making a meaningful difference in their lives.

## CONSIDERATIONS FOR EARLY-STAGE SETTING

Integrating early palliative and supportive care has demonstrated economic benefits, particularly through in-hospital referrals. These benefits could be extended to surgical settings by incorporating them in the ‘Enhanced Recovery After Surgery’ (ERAS) protocols [9], which standardize patient support based on individual needs, values and psycho-social circumstances. Tailored approaches reduce hospital stays while ensuring patients receive appropriate care when symptoms become intolerable.

Ultimately, patients themselves are the best guides for improving their treatment experience. By continuously monitoring their QoL and involving them in care decisions, we can ensure timely and active support, avoid unnecessary healthcare resource utilization and prevent treatment discontinuation. Survivorship is about more than being disease-free—it is about empowering patients to live well, even in the aftermath of cancer.
